# Comparative analysis of hemoglobin, potassium, sodium, and glucose in arterial blood gas and venous blood of patients with COPD

**DOI:** 10.1038/s41598-024-55992-9

**Published:** 2024-03-02

**Authors:** Sitian Tang, Zhu Mei, Dongmei Huang, Ling Liu, Lunyu Yang, Dan Yin, Liyi Hu

**Affiliations:** People’s Hospital of Chongqing Liang Jiang New Area, No. 199 Ren Xing Road, Chongqing, 401135 People’s Republic of China

**Keywords:** Biological techniques, Biotechnology, Diseases, Health care, Medical research

## Abstract

The study aims to assess the accuracy of the arterial blood gas (ABG) analysis in measuring hemoglobin, potassium, sodium, and glucose concentrations in comparison to standard venous blood analysis among patients diagnosed with chronic obstructive pulmonary disease (COPD). From January to March 2023, results of ABG analysis and simultaneous venous blood sampling among patients with COPD were retrospectively compared, without any intervention being applied between the two methods. The differences in hemoglobin, potassium, sodium, and glucose concentrations were assessed using a statistical software program (R software). There were significant differences in the mean concentrations of hemoglobin (*p* < 0.001), potassium (*p* < 0.001), and sodium (*p* = 0.001) between the results from ABG and standard venous blood analysis. However, the magnitude of the difference was within the total error allowance (TEa) of the United States of Clinical Laboratory Improvement Amendments (US-CLIA). As for the innovatively studied glucose concentrations, a statistically significant difference between the results obtained from ABG (7.8 ± 3.00) mmol·L^−1^ and venous blood (6.72 ± 2.44) mmol·L^−1^ was noted (*p* < 0.001), with the difference exceeding the TEa of US-CLIA. A linear relationship between venous blood glucose and ABG was obtained: venous blood glucose (mmol·L^−1^) =  − 0.487 + 0.923 × ABG glucose (mmol·L^−1^), with R^2^ of 0.882. The hemoglobin, potassium, and sodium concentrations in ABG were reliable for guiding treatment in managing COPD emergencies. However, the ABG analysis of glucose was significantly higher as compared to venous blood glucose, and there was a positive correlation between the two methods. Thus, a linear regression equation in this study combined with ABG analysis could be helpful in quickly estimating venous blood glucose during COPD emergency treatment before the standard venous blood glucose was available from the medical laboratory.

## Introduction

Chronic obstructive pulmonary disease (COPD) is a prevalent disease in respiratory and critical care unit, especially among the elderly. Besides, COPD greatly affects patients' quality of life and increases the burden on their families^1^. According to statistics, more than 3 million people die of COPD worldwide each year^2^. A study in 2020 revealed that COPD was one of the third leading causes of death worldwide^3^. Therefore, a timely diagnosis and assessment of COPD can assist clinicians to standardize the treatment of patients^4–6^.

Arterial blood gas (ABG) analysis is a commonly used tool for monitoring patients with COPD^7^. It provides clinicians with information on the patient's acid–base balance, partial pressures of oxygen and carbon dioxide, as well as levels of hemoglobin, potassium, sodium, and glucose simultaneously. This allows clinicians to quickly assess the oxygenation, internal environment, and electrolyte balance in COPD patients, and to determine appropriate initial treatment^8,9^.

Due to the fact that most COPD patients are elderly^10,11^, simultaneous collection of arterial and venous blood can exacerbate their distress and increase the risk of complications such as bleeding, hematoma, infection, nerve injury, and finger ischemia^12,13^. Additionally, the extra blood collection places a strain on medical laboratories and nursing staff^14^. If ABG parameters could replace venous blood measurements of hemoglobin, potassium, sodium, and glucose, clinicians could more readily provide initial management for a variety of conditions until conclusive laboratory results become available. This approach will accelerate diagnosis and treatment while also reducing discomfort experienced by elderly patients and improving the doctor–patient relationship^15–17^.

Sinan Yilmaz found significant differences in hemoglobin, potassium, and sodium measurements between ABG analysis and venous blood testing in the Intensive Care Unit^18^. Potassium and sodium measurements should be based on venous blood testing; Anunaya Jain concluded that ABG results for potassium and sodium couldn't substitute for venous blood tests after comparing results from arterial and venous blood in the emergency department^19^; However, Jérôme Allardet–Servent and other scholars determined that arterial blood analysis showed no significant difference in potassium and sodium measurements compared to venous blood tests, and ABG could be analyzed instead of venous blood tests^20^; Moreover, according to the United States of Clinical Laboratory Improvement Amendments (US-CLIA), ABG analysis can accurately replace venous blood tests as a clinical reference if the results remain within the total error allowance^21^.

The study was conducted between January and March 2023 in our hospital’s respiratory and critical care unit, where 165 patients diagnosed with COPD were selected. The aim of the study was to investigate the levels of hemoglobin, potassium, and sodium concentration in ABG analysis and compare them with levels in venous blood parameters. The differences between arterial and venous blood levels were analyzed. Additionally, the study innovatively analyzed the difference between arterial and venous blood glucose levels. This approach by our team lays a firm basis for clinical diagnosis and treatment.

## Results

A total of 165 patients were included in this study for analysis of hemoglobin, potassium, and sodium, of which 48 patients were included in the comparison of glucose analysis. Table 1 summarized the mean differences in hemoglobin, potassium, sodium, and glucose concentrations between the two modalities (ABG and venous blood samples).

**Table 1:** Analysis of hemoglobin, potassium, sodium, and glucose concentrations in arterial blood gas and venous blood (x ± s).

Item	Arterial blood gas	Venous blood	Difference^△^	*t*	*p*	*R*^2^	Equation
Hb/(g·L^−1^)	139.71 ± 19.53	131.09 ± 18.45	8.62 ± 9.69	11.284	< 0.001	0.871	18.806 + 0.922x
K^+^/(mmol·L^−1^)	3.72 ± 0.44	3.91 ± 0.43	− 0.19 ± 0.21	11.891	< 0.001	0.888	0.111 + 0.922x
Na^+^/(mmol·L^−1^)	137.57 ± 3.52	138.07 ± 3.58	− 0.50 ± 1.95	3.274	0.001	0.848	22.421 + 0.834x
Glu (mmol·L^−1^)	7.80 ± 3.00	6.72 ± 2.44	1.08 ± 1.43	5.263	< 0.001	0.882	− 0.487 + 0.923x

△Arterial blood gas concentration minus venous blood concentration; Hb: hemoglobin; K^+^: potassium; Na^+^: sodium; Glu: glucose.

### Hemoglobin (Hb)

**Figure 1:**
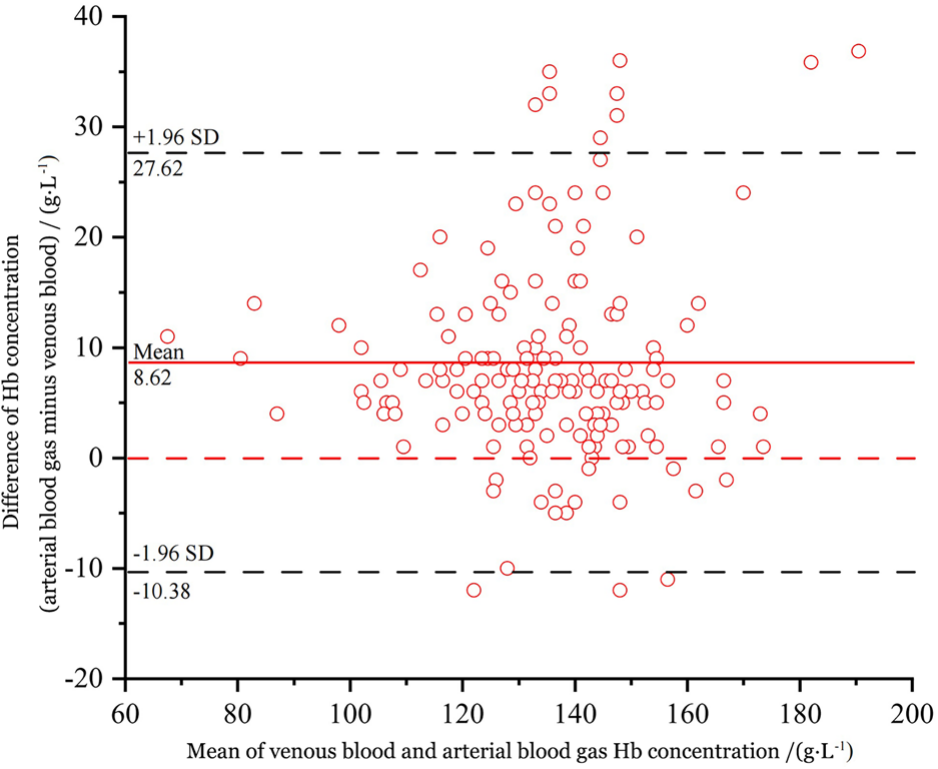
Bias plot of the hemoglobin concentration.

Hemoglobin (Hb) concentrations were determined using ABG and venous blood tests respectively, and the results were shown in Table 1. The mean hemoglobin concentration in ABG was (139.71 ± 19.53) g·L^−1^, while the mean concentration in venous blood was (131.09 ± 18.45) g·L^−1^, and there was a significant difference of (8.62 ± 9.69) g·L^−1^ in the mean concentration of hemoglobin between the two (*p* < 0.001, paired *t* test, t = 11.284). Figure 1 depicted the Bland–Altman plot of arterial and venous hemoglobin.

### Potassium (K^+^)

**Figure 2:**
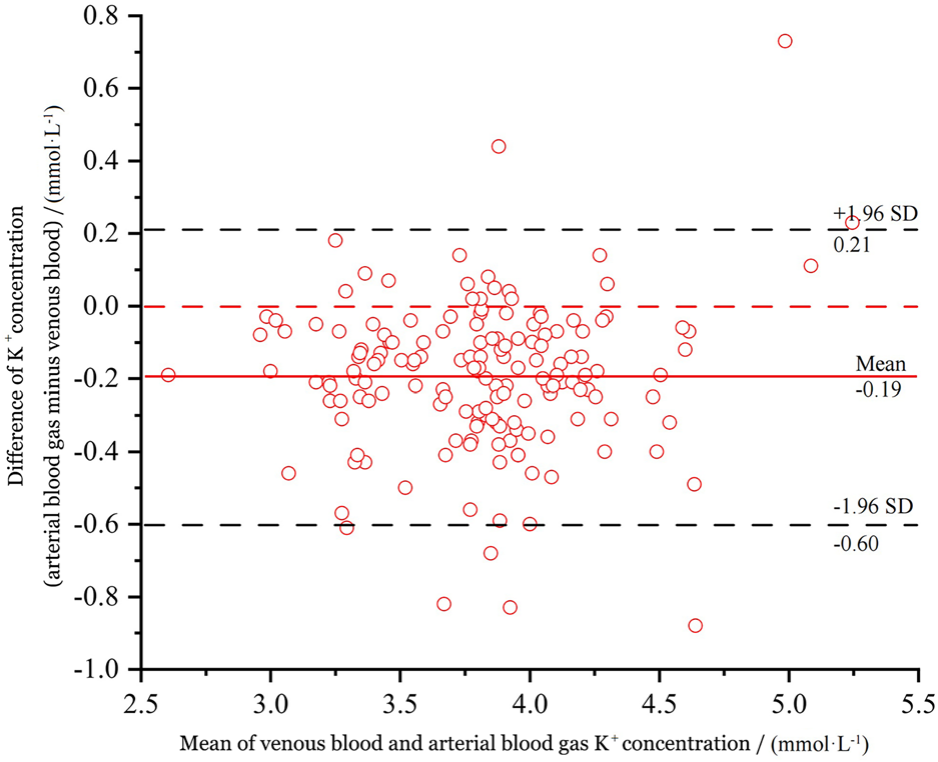
Bias plot of the potassium concentration.

Potassium (K^+^) concentrations were compared between two analyzers. The overall mean difference was (− 0.19 ± 0.21) mmol·L^−1^, which was statistically significant (*p* < 0.001, paired *t* test, t = 11.891). The mean values of potassium concentrations in ABG and venous blood were (3.72 ± 0.44) mmol·L^−1^ and (3.91 ± 0.43) mmol·L^−1^ respectively, with higher values measured in venous blood than in ABG. The corresponding Bland–Altman plot for potassium concentrations in arterial and venous blood was shown in Fig. 2.

### Sodium (Na^+^)

**Figure 3:**
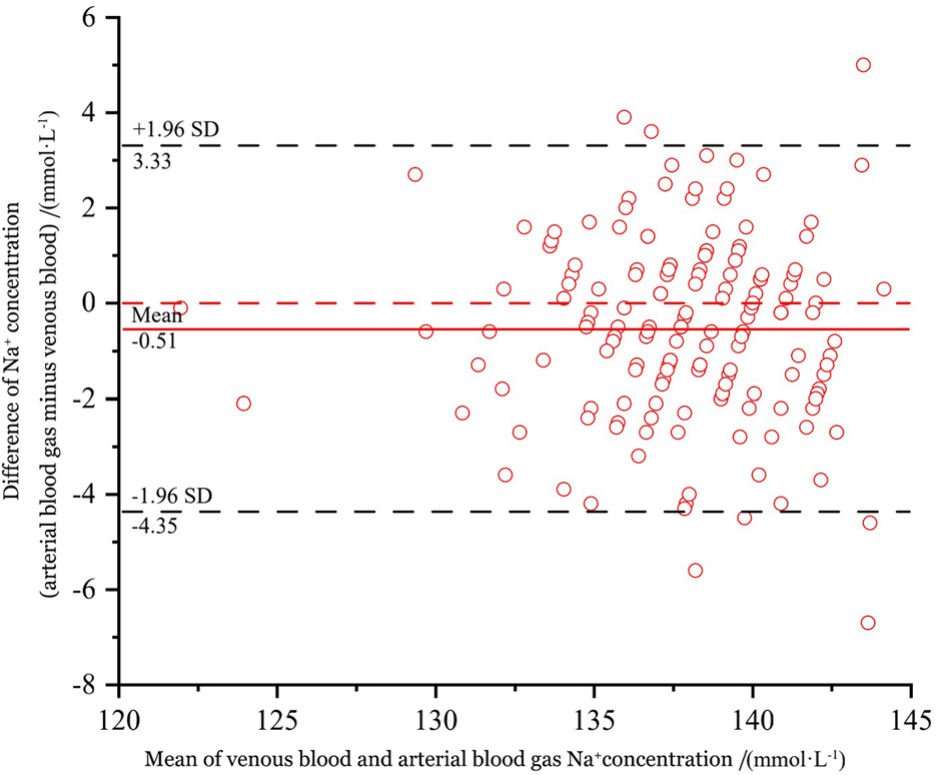
Bias plot of the sodium concentration.

Sodium (Na^+^) levels in ABG and venous blood were analyzed using two different analyzers. The overall mean difference in sodium concentrations between the two analyzers was (− 0.50 ± 1.95) mmol·L^−1^, with ABG having a mean sodium concentration of (137.57 ± 3.52) mmol·L^−1^ and venous blood having a mean sodium concentration of (138.07 ± 3.58) mmol·L^−1^ (*p* = 0.001, paired *t* test, t = 3.274). Figure 3 shows the Bland–Altman plot corresponding to sodium.

### Glucose (Glu)

**Figure 4:**
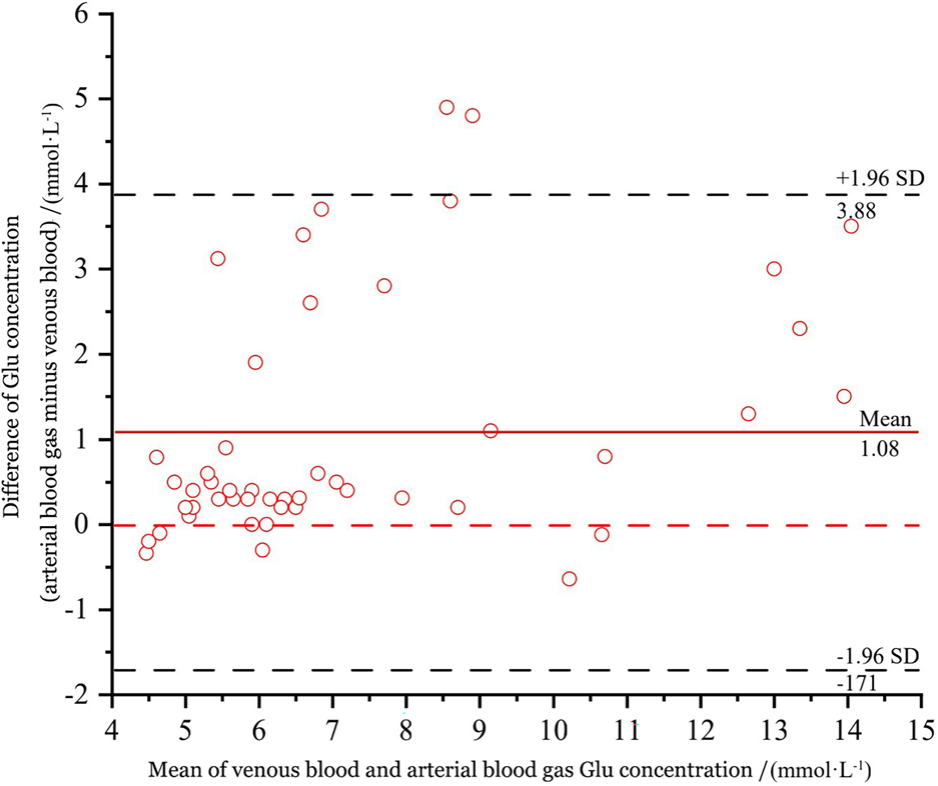
Bias plot of the glucose concentration.

**Figure 5:**
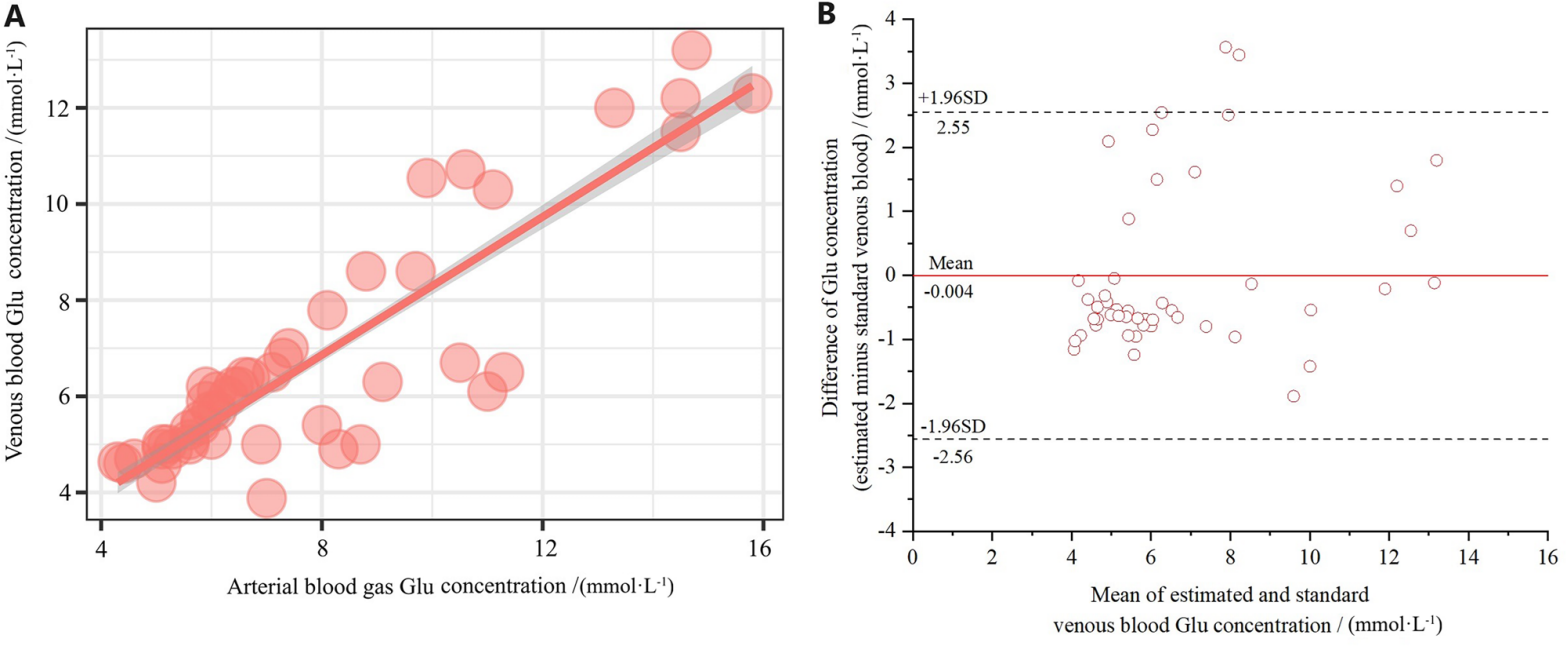
(**A**) Scatter plot of the glucose concentration with Deming fit. (**B**) Bias plot of estimated and standard venous glucose concentrations.

A total of 48 patients were included in the analysis of arterial and venous glucose (Glu) differences because fewer patients had arterial and venous blood glucose collected simultaneously. On comparing glucose concentrations, there was a significant mean difference of (1.08 ± 1.43) mmol·L^−1^ between the ABG analyzer and the venous laboratory analyzer (*p* < 0.001, paired *t* test, t = 5.263). Glucose concentrations were (7.80 ± 3.00) mmol·L^−1^ for ABG and (6.72 ± 2.44) mmol·L^−1^ for venous blood. The Bland–Altman plot for glucose was illustrated in Fig. 4. According to the Deming regression shown in Fig. 5A, the linear regression equation was obtained: venous blood glucose (mmol·L^−1^) =  − 0.487 + 0.923 × ABG glucose (mmol·L^−1^). The correlation coefficient (R^2^) between the two types of glucose measurements was 0.882. Next, to verify the consistency of the estimated glucose concentration obtained from the above linear regression with the standard venous glucose concentration, Bland–Altman analysis was also carried out (Fig. 5B).

## Discussion

This study was conducted to assess the concordance between ABG and standard venous laboratory analyzer estimations of hemoglobin, potassium, sodium, and glucose concentrations in patients in the respiratory and critical care unit. Since there was limited literature comparing the differences between the two cohorts, the aim of this study was to determine whether physicians could be confident in the results of these two modalities being interchangeable in the diagnosis and management of COPD patients.

The study demonstrated a significant difference in hemoglobin concentrations between venous and ABG samples. Of the 165 samples, 92% (152/165) fell within the 95% LOA (− 10.38, 27.62, *p* < 0.001), while more than 5% values fell outside the limits. Poor agreement was observed between venous and ABG hemoglobin measurements. The data presented in Table 1 show a mean difference of (8.62 ± 9.69) g·L^−1^ in hemoglobin concentrations between arterial and venous samples, with a clinical error variability of 6.6%. However, the mean biases did not exceed the acceptable biases determined by the US-CLIA (7%). Consequently, ABG hemoglobin concentrations could be used as a quick determination of venous blood hemoglobin concentrations by respiratory and critical care unit physicians for emergency COPD evaluation until venous hemoglobin measurements are available.

According to the findings presented in Table 1, the potassium concentration in venous blood was slightly higher than ABG, and showed statistically significant differences, which is consistent with Zhang's research^22^. 94% (156/165) values were within the 95% LOA (− 0.60, 0.21, *p* < 0.001), with an out-of-bounds percentage of 6%, and the two measurements were not in good agreement. The mean difference between the measured venous and ABG potassium concentrations was − 0.19 mmol·L^−1^ but within the TEa range of ± 0.5 mmol·L^−1^. Therefore, while there was some variability between the measurements, it was still within an acceptable range. Therefore, it may be considered that ABG potassium concentration could be used for prompt assessment before the arrival of laboratory results.

Venous blood potassium concentrations were found to be slightly higher than ABG potassium concentrations due to several reasons. First, there are inherent differences between ABG and venous blood. Second, differences in specimen collection tubes could also contribute to this variance. Specifically, ABG analysis was performed with sodium heparin as the anticoagulant, which diluted the arterial blood specimen, resulting in lower arterial potassium levels compared to those of the venous blood. Lastly, the specimen types used for testing are different. ABG potassium analysis was performed on whole blood, while venous blood potassium testing required blood coagulation and centrifugation. As blood cells contain a large number of potassium ions, hemolysis during blood collection or other processes could cause these ions to enter the blood, resulting in elevated venous blood potassium levels. To improve accuracy, specimens should be sent for testing as quickly as possible after clinical collection.

This study found a statistically significant difference between the measurements of sodium concentration in venous and ABG. The study observed that 158 out of a total of 165 data points (96%) were within the 95% LOA of − 4.35 to 3.33 with a significance level of *p* = 0.001. Only 4% of the data points were outside the limits of agreement, indicating satisfactory agreement between the two methods of measurement. Moreover, the mean difference observed was − 0.5 mmol·L^−1^. According to the US-CLIA guidelines, a difference of ± 4 mmol·L^−1^ from the gold-standard calibration solution was acceptable for sodium concentration. Thus, using ABG sodium concentrations for estimating venous blood sodium concentrations during the acute assessment is plausible.

An accurate glucose value was vital in the identification of acute presentations such as COPD combined with diabetes, especially in the management of maintaining strict glucose control in the critically ill. The current investigation discovered a statistically significant mean difference between the two modalities. ABG glucose concentrations were higher than those of venous blood glucose. The percentage of data within the 95% LOA (− 1.71, 3.88, *p* < 0.001) bounds of agreement was 95.8% (46/48), with a strong agreement between the two measurements, corresponding with the findings of a Canadian investigation^23^. The mean difference between ABG and venous blood glucose concentrations was 1.08 mmol·L^−1^, surpassing the US-CLIA TEa for glucose of ± 0.33 mmol·L^-1^. Hence, ABG glucose concentration was unsuitable for a quick examination of venous glucose values. Deming regression showed a positive correlation between ABG and venous blood glucose concentrations, with a correlation coefficient R^2^ = 0.882.

In addition, an estimated venous blood glucose concentration could be obtained from the ABG glucose concentration, using the formula “venous blood glucose (mmol-L^−1^) =  − 0.487 + 0.923 × ABG glucose (mmol-L^−1^)”. Bland–Altman analysis was also performed to compare the standard venous blood glucose concentrations and the estimated concentrations. The 95% LOA (− 2.56, 2.55, *p* < 0.001) bounds of agreement were satisfied by 95.8% (46/48), indicating strong consistency between the estimated and the standard venous blood glucose. Although the mean difference between them was only − 0.004 mmol·L^-1^, the Bland Altman's 95% LOA (-2.56, 2.55) mmol·L^-1^ for glucose was wider than the US-CLIA TEa of ± 0.33 mmol·L^-1^, which was not clinically acceptable. Hence, the estimated mean concentration of venous blood glucose obtained according to the linear regression equation was only able to reflect the mean value of the standard venous blood glucose to a certain extent, which could help clinicians make a quick and preliminary judgment of the blood glucose concentrations for rapid assessment of COPD patients before standard venous blood glucose was available from the medical laboratory^24^. However, it was noteworthy that estimating blood glucose couldn't completely substitute for standard venous blood glucose, thus final results should be still subjected to the laboratory venous blood glucose concentrations.

The following are the causes for the large discrepancy in arterial and venous blood glucose levels. First of all, as the name says, ABG glucose is taken from arterial blood, and venous blood glucose is taken from peripheral venous blood. There is a natural distinction between the two methods. Moreover, carbohydrate is absorbed into the blood and first enters the artery. After digestion and absorption, it is gradually transported to the capillaries, and gradually flows back to the vein, causing the arterial glucose higher than the venous blood glucose level. In addition, due to the rapid arterial blood flow, arterial glucose fluctuates a lot and is not very stable, in contrast to venous blood glucose, which is more consistently stable. At the same time, this is also the reason why venous blood is commonly used in clinical glucose analysis.

In terms of limitations, the study did not assess whether or not the extent of the difference had an effect on patient outcomes. Therefore, it is suggested that this be taken into account in future research. Additionally, only one ABG machine and one standard venous analyzer were employed, thus subsequent research should evaluate the agreement across several machines and compare each one to standard venous analyzers separately.

## Conclusion

To summarize, given that most COPD patients are elderly, it is advisable to use ABG hemoglobin, potassium, and sodium to assess venous blood in order to minimize discomfort caused by multiple blood collections from the elderly. In addition, the combined use of ABG and the linear regression equations for glucose in this study also helped physicians assess venous blood glucose to rapidly evaluate COPD patients' condition and treatment, before the availability of standard venous blood concentrations from laboratory venous blood analyzers.

## Methods

### General information

This observational study was conducted between January and March 2023, and it involved patients who were diagnosed with COPD and admitted to the respiratory and critical care unit of our hospital. A total of 165 patients (122 males and 43 females, aged 56 to 101) whose ABG analysis and venous blood samples were collected at the same time and tested for hemoglobin, potassium, and sodium levels were included in the study. Additionally, the glucose levels of 48 patients (38 males and 10 females, aged 56 to 101) were innovatively included in the comparison. Hemoglobin, potassium, sodium, and glucose concentrations in the patient’s ABG analysis and venous blood were measured, and a database was established. The study was approved by the Medical Ethics Committee of the People's Hospital of Chongqing Liang Jiang New Area and was conducted in line with the relevant guidelines and regulations. All subjects provided written informed consent.

### Instruments and reagents

The Siemens blood gas analyzer was used to examine all ABG samples, and Siemens Atellica Healthcare Laboratory Analyzer and Sysmex XN-1500 Automatic Blood Cell Analyzer were utilized to analyze venous samples. Each device underwent timely and routine calibration consistent with corporate standards. Both machines measured and analyzed different parameters, including hemoglobin, potassium, sodium, and glucose concentrations. The parameters' outcomes were retrieved from the hospital information system (Laboratory Information System, LIS), and ABG results for each parameter were compared directly with venous blood results.

### Experimental methods

Upon receiving specimens from the respiratory and critical care unit, arterial blood with heparin anti-coagulation underwent testing using the Siemens blood gas analyzer to determine hemoglobin, potassium, sodium, and glucose concentrations. Immediately, venous blood with heparin anti-coagulation underwent centrifugation at 3000 r/min for 5 min. Measured venous serum potassium, sodium, and glucose concentrations on Siemens Atellica Healthcare Laboratory Analyzer. Venous blood with EDTA-K2 anti-coagulation underwent measurement using the XN-1500 Automatic Blood Cell Analyzer for detecting venous blood hemoglobin concentration. All the staff who used these instruments underwent training and certification for the instruments' usage and upkeep, and all the procedures were in accordance with the standard operating procedures of the medical laboratory^13^. The experimental protocols were approved by the People's Hospital of Chongqing Liang Jiang New Area.

### Statistical methods

The data in the study were analyzed using the R software, and the measures were presented as mean ± standard deviation (x ± s). The differences between ABG analysis and venous hemoglobin, potassium, sodium, and glucose concentrations were analyzed using a paired t-test. A statistically significant difference was considered at *p* < 0.05. The 95% limits of agreement (95% LOA) for the measured data were calculated using the Bland–Altman plot. To assess the clinical significance of the differences, the total error allowance (TEa) of US-CLIA was used as the basis^12^. The TEa was ± 7% for hemoglobin, ± 0.5 mmol·L^-1^ for potassium, ± 4 mmol·L^-1^ for sodium, and ± 0.33 mmol·L^-1^ for glucose. Besides, the correlation coefficients between ABG analysis and venous blood hemoglobin, potassium, sodium, and glucose were calculated, and the linear regression equations were established using Deming regression.

## Data Availability

The datasets generated during and/or analyzed during the current study are available from the corresponding author on reasonable request.
